# Viburnum Prunifolium

**Published:** 1882-12

**Authors:** 


### VIBURNUM PRUNIFOLIUM.

Dr. O. E. Herrick, in commenting upon the occasional toxic effects of viburnum prunifolium dryness of the mouth, dimness of vision, dizziness, etc.), says: "Next to ergot I place more dependence upon viburnum in the treatment of diseases of females than any other agent for internal administration. Its action is not limited to the prevention of miscarriage by any means, but it is alike applicable in cases of uterine engorgement, ovarian irritation, and numerous other conditions of the female sexual organs."—*Obstetric Gazette.*

